# Patient-Perceived Health System Responsiveness of the Epilepsy Management Project in Rural China during the Period of COVID-19

**DOI:** 10.3390/healthcare10050799

**Published:** 2022-04-25

**Authors:** Lulu Qin, Si Chen, Xianglin Feng, Bangan Luo, Yiwei Chen

**Affiliations:** 1 Department of Social Medicine and Health Management, School of Medicine, Hunan Normal University, Changsha 410013, China; powerestlulu@163.com (L.Q.); miraitowasi@163.com (S.C.); xianglin9310000@163.com (X.F.); 2 Department of Mental Health, Brain Hospital of Hunan Province, Changsha 410007, China; 3 Department of Social Medicine and Health Management, Xiangya School of Public Health, Central South University, Changsha 410078, China; 4 Department of Neurology, Xiangya Third Hospital, Central South University, Changsha 410017, China

**Keywords:** responsiveness, HSR, epilepsy patients, the Epilepsy Management Project, COVID-19

## Abstract

Health system responsiveness (HSR) measures the experience of health-system users in terms of the non-clinical domains of the health system, which has been regarded as the three major goals of health performance evaluation. Good HSR may promote the use of health services and ultimately the health of patients. However, the HSR has not been measured as the main goal of the Epilepsy Management Project (EMP) in rural China. This study aims to evaluate the levels and distributions of the patient-perceived HSR of the EMP in rural China during the period of COVID-19 and identify its relevant factors so as to provide advice on the improvement of further strategies. Based on the key informant survey (KIS) of responsiveness from the World Health Organization proposal, we conducted a cross-sectional survey of 420 epilepsy patients selected proportional randomly from seven rural areas in the Hunan province of China in 2021. Eight domains of patients-perceived HSR were assessed by face-to-face interview. The overall HSR scored at a fairly “good” level of 8.3 (8.3 out of a maximum of 10.0). During the COVID-19 period, the scores of responsiveness domains were highest at 8.66 to 8.93 in “confidentiality”, “dignity” and “choice of providers”, while lowest at 8.38 to 8.53 in “prompt attention”, “social support” and “basic amenities”. The representative responsiveness equality index (REI) was 0.732, indicating the moderately balanced distributions of responsiveness of the EMP in rural China. Female, old age, and low education were significantly related to the lower HSR scores of rural EMP (*p* < 0.05). The HSR of EMP in rural China was fairly good. However, measures to improve the patient-perceived HSR are still needed, especially including better service, higher social support, and more comfortable medical environments.

## 1. Introduction

Epilepsy, the second most common disease in neurology, has more than nine million patients in China, with a high prevalence and incidence rate [1]. Epilepsy is a chronic recurrent paroxysmal transient brain dysfunction syndrome, which is mainly characterized by recurrent epileptic seizures caused by abnormal discharge of brain neurons. Due to the neurobiochemical, cognitive, psychological, and social consequences caused by epilepsy, epilepsy has brought a serious burden to patients, their families, and society, which has become an important public health and social problem. Epileptic patients need long-term use of antiepileptic drugs (AEDs) to control the disease, and their sudden withdrawal from drugs might often lead to the recurrence of epilepsy, which brings noteworthy challenges to patients and the health service system [1].

Due to different economic levels, medical and health conditions, and patients’ cognition of epilepsy in various areas, there are obvious differences in the treatment of epilepsy among different regions. Previous studies showed that there were a large number of epileptic patients that have never received antiepileptic treatment or treatment irregularly [2,3,4]. The World Health Organization (WHO), the International Bureau for Epilepsy (IBE), and the International League Against Epilepsy (ILAE) have reported that the treatment gap for epilepsy in developing countries varied from 60% to 98% [3,4,5,6]. In China, the treatment gap for active epilepsy was high [5]. Recent research in rural China showed that epileptic patients who have never received antiepileptic treatment accounted for 46.73% [6]. In addition, among the epileptic patients receiving antiepileptic treatment, there were 24.2–50.0% of them did not receive treatment or with poor treatment compliance [6,7]. Based on the traffic conditions, economic conditions, and shame among epilepsy patients in rural areas, therefore, the treatment of epilepsy patients in rural areas needs to be paid more attention.

Health system responsiveness (HSR) measures the experience of health-system users in terms of the non-clinical domains of the health system. At the first time proposed by the WHO in 2000, the HSR has been regarded as the three major goals of health performance evaluation [8]. HSR refers to the non-financial domains of health service and non-clinical health domains, including eight domains of dignity, confidentiality, autonomy, prompt attention, the quality of basic amenities, social support, and the choice of provider [9]. Numerous studies have indicated that good HSR may promote the use of health services and the health ultimately of patients [10,11].

Recorded in many studies, HSR and its related concepts have been operationalized as a measurable construct in multiple surveys and studies, especially among several non-communicable diseases (NCDs) [12]. Responsiveness increases patient satisfaction with the health system, which in turn leads to higher utilization of health care services [13,14]. A study from Ethiopia reported that the HSR was independently related to the satisfaction of HIV care [15], while another study found that the HSR was associated with increasing visit adherence [16]. Some domains of HSR were identified as priority areas to improve the responsiveness of health services, such as prompt attention, communication, and autonomy [17,18]. Previous studies have shown that HSR is different in terms of socioeconomic status (SES), areas, social environment, and populations [19]. However, there has been no study that applied the HSR concept and its determinants thoroughly for the evaluation of the epilepsy health system.

Good HSR is the key to patients’ adherence to treatment, especially during the special periods like the COVID-19 epidemic. During the COVID-19 epidemic period, epileptic patients are confronted with additional risks, especially in the rural areas due to their lack of health resources. Patients with epilepsy have to face difficulties in seeking and utilizing health services under the measures of isolation and restriction during the COVID-19 epidemic, which may even result in their cutting off the drug. At the same time, anxiety, panic, and other psychological and physiological reactions of epileptic patients are increased with the spread of the epidemic, which may result in the increasing risk of epileptic seizure [20,21]. Consequently, the performance of HSR is more important when meeting some urgent situations such as the period of the COVID-19 epidemic. Therefore, it is necessary and urgent to understand and improve the HSR during emergency situations.

In 1977, WHO together with ILAE and IBE, launched a “global campaign against epilepsy (GCAE)” aimed at improving global awareness, treatment, and services for epilepsy. In 1999, the WHO, GCAE experts, and Chinese experts formulated the “Plan of Epilepsy Management Project in rural China”, which was an important part of GCAE. Taking advantage of China’s existing primary health care system, the Epilepsy Management Project (EMP) in rural China has been launched in 2008, which aims to increase the treatment rate of the epileptic patient by providing epilepsy patients with free antiepileptic drugs regular follow-ups. The EMP was based on three approaches; reducing patients’ out-of-pocket payments, improvement of the equity in access to health services, and promoting the quality of services rendered [22]. Studies have assessed the success of EMP in different areas such as health promotion and costs [23,24]. However, the HSR has not been measured as the main goal of the Epilepsy Management Project in rural China. Thus, to identify problems affecting the Chinese health system and those actions that will lead to its improvement.

Considering the designers of the EMP have considered responsiveness as one of the main objectives of the plan, this study aims to evaluate the levels and distributions of the patient-perceived HSR of the EMP in rural China during the period of COVID-19, and its relevant factors so as to provide the advice on the improvement of further strategies. Given the importance of responsiveness as one of the performance indicators of the health system, this study will bridge the research gap by exploring the patients-perceived HSR of the EMP, and provide the new horizons for research in the area of epilepsy management to improve the service innovation capability of the EMP. In addition, the article concludes with the study’s qualification, plus some practical implications, suggesting future research directions.

## 2. Materials and Methods

### 2.1. Study Design and Sample Size Calculation

This is a cross-sectional study, and we conducted this study in rural areas of Hunan province, China. Hunan, located in the south-central of China, was one of the first areas to implement the Epilepsy Management Project. At the end of 2020, there are a total of 7 rural areas of Hunan province implementing the Epilepsy Management Project.

Sample size estimation was calculated using the formula for cross-sectional studies: α = 0.05, *n* = u_α/2_^2^P(1 − P)/d^2^, where u = 1.96 when α = 0.05, P is the prevalence of the poor evaluation rate of HSR (which is 47% in this study), and d is admissible error (which was 5 % here). The theoretical sample size was 421 which includes an extra 10% to allow for participants lost during the study.

### 2.2. Study Population and Procedures

According to the Epilepsy Management Project, the service objects of the EMP are the participants diagnosed with convulsive epilepsy who are aged 2 years and above. The Epilepsy Management Project provides these epilepsy patients with free antiepileptic drugs and regular follow-ups to improve their disease outcomes. 

Thus, in this study, we selected epilepsy patients as our respondents if they met the inclusion criteria: a. epilepsy patients have participated in the EMP for more than 12 months; b. epilepsy patients who were willing to participate in this investigation. Exclusion criteria: a. epilepsy patients outside of the EMP; b. epilepsy patients who were not suitable to participate in this study were excluded, such as severe mental disorders and consciousness disorders.

We used the proportional sampling method according to the unique identifier number of patients in the Epilepsy Management Project to select a representative sample of the rural epileptic patients between January and September 2021. Among the 7 rural areas of Hunan province implementing the Epilepsy Management Project at the end of 2020, there were a total of 4260 epilepsy patients meeting the inclusion criteria in the EMP in Hunan province (each area covering epileptics varied from 300 to 700, respectively). Then, 10% of rural epileptic patients were randomly selected from each rural area of the Epilepsy Management Project.

Participants were interviewed face to face by trained interviewers after giving written, informed consent. If the participant was illiterate or under the age of 18, the consent was signed by their guardians, and those participants were investigated with the help of their guardians. Finally, 426 participants were selected, and 420 of them have completed this, while 6 of the participants did not complete this investigation. 

### 2.3. Data Collection and Measures

The self-designed questionnaire was based on the KIS (key informant survey) questionnaire from the WHO, which had been modified for our study purpose. The questionnaire contains 2 parts: socio-demographic information and patient-perceived health system responsiveness.

### 2.4. Socio-Demographic Information

Socio-demographic information includes age, gender, marital status (stable, unstable), education, and occupation (farmer, unemployed, student, else). Education was assessed by asking participants to select their highest level of education from the following choices: below primary school, primary school, middle school, high school, and above. Other information includes: type of health insurance, duration of disease (years), treatment cost (Renminbi, RMB), type of hospital visited, annual income, etc. In this study, stable marital status was defined as marriage; while unstable marital status included divorced, widowed or widowed, never married, unmarried cohabitation, and so on. In addition, RMB is the legal tender of the people’s Republic of China.

### 2.5. Patient-Perceived Health System Responsiveness

The key informant survey (KIS) questionnaire designed by WHO was used to survey the patients-perceived HSR in this study, which has been proved to be an effective measurement tool to collect responsiveness data in a short time and is widely used [25,26,27,28]. In China, the KIS questionnaire has been translated into Chinese and is widely used in many regions across China, which has been proven to be of good reliability and validity [29]. According to WHO, the HSR comprises two parts: “Respect for Persons” and “Client Orientation”. (1) “Respect for Persons” includes dignity (being treated with respect), autonomy (involvement in decision-making), confidentiality (personal data is kept confidential), and communication (the provider listens carefully and explains things clearly); (2) “Client Orientation” includes prompt attention (getting fast care in emergencies, short waiting times), social support (access to social networks during inpatient care), basic amenities (cleanliness of the facility, seating, fresh air), choice of providers (possibility to choose between different providers) [25].

Based on the KIS, participants were asked to rate their experience of contact with the EMP health services. The Likert-4 quantification method was used to evaluate the 8 domains at four levels (always, usually, sometimes, and never). Meanwhile, each domain was scored using a 10-point scoring system in which Best = 10 and Worst = 0. We calculated each domain score using averages. The weighted mean score of each responsiveness domain was then determined, as recommended by the WHO [8]. The higher the score, the better the responsiveness. 

**Figure 1:**
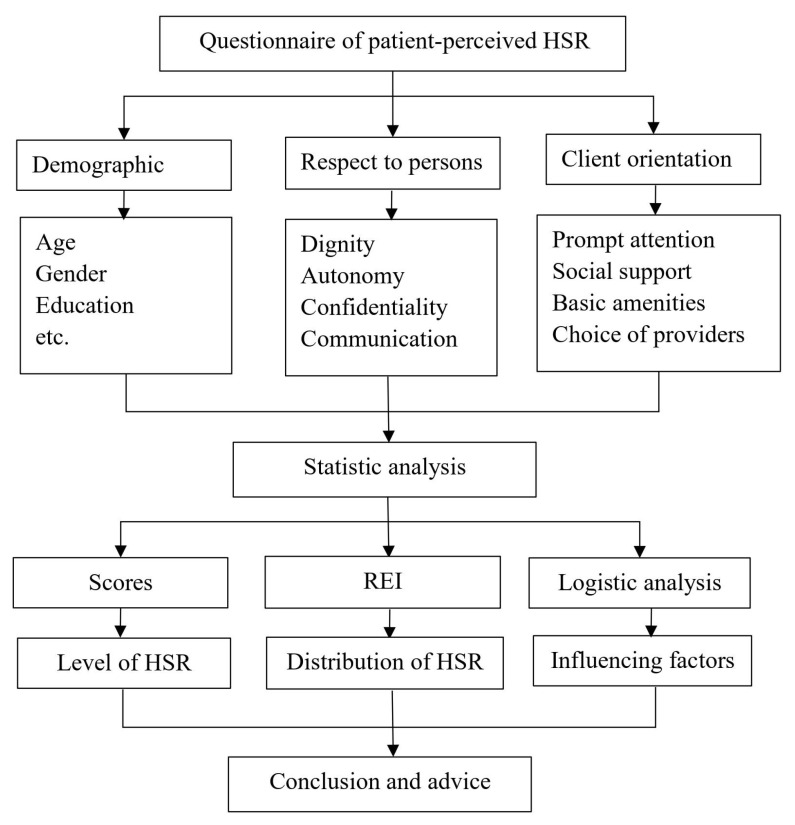
The framework of responsiveness evaluation.

The patient-perceived health system responsiveness questionnaire was shown in Figure 1.

### 2.6. Statistic Analysis

Data were analyzed using SPSS V.20.0 (SPSS/IBM, Armonk, NY, USA). Descriptive statistical methods were used such as the percentage, and mean ± SD. Non-parametric tests were used because the distribution of the score of the patients-perceived HSR was non-Gaussian.

#### 2.6.1. Scores of Responsiveness Levels

The WHO-recommended formula was used to calculate the responsiveness scores, which are equal to the sum of the weighted scores of the eight elements. The corresponding weight coefficients for each of the eight elements mentioned above were 0.125 (dignity), 0.125 (autonomy), 0.125 (confidentiality), 0.125 (communication), 0.20 (prompt attention), 0.15 (social support), 0.10 (basic amenities), and 0.05 (choices of providers), respectively [2]. The *t*-test, one-way ANOVA analyses, or Kruskal–Wallis test were used to explore differences in patient-perceived HSR scores among patients with different characteristics.

#### 2.6.2. Responsiveness Distribution

According to the WHO, the distribution of patient-perceived HSR was evaluated by the responses from the disadvantaged groups (female, elderly, low education, low income, farmer) [8] and the responsiveness equality index (REI) in this study. The closer the reactivity equality index is to 1, the more fair the responsiveness distribution is REI = 1-RIS (Reactivity inequity score), RIS = KⅡp × Pp (KⅡp: the number of disadvantaged people in the total participants, Pp: the rate of disadvantage people in the total participants) [8].

#### 2.6.3. Risk Factors for Low HSR among Rural China Epileptic Patients in Epilepsy Management Project

Binary logistic regression analysis. Binary logistic regression analysis was performed to identify risk factors for low HSR scores among rural China epileptic patients. Odds ratio (OR) and corresponding 95% confidence interval (95% CI) were used to evaluate the relationship between independent variables and level of HSR. The median value of the score of HSR was used to definite groups. The score of HSR was selected as the dependent variables and classified into group 1 (score < 8) and group 2 (score ≥ 8). Gender, age (1 = 39 years old and below, 2 = 40–59 years, 3 = 60 years and above), marital status (1 = stable and 2 = unstable), occupation (1 = farmer, 2 = employment, 3 = unemployment, 4 = student), education (1 = primary school and below, 2 = middle school, 3 = high school and above), number of recurrent seizures in the past year (1 = 0, 2 = 1–2, 3 = 3–4, 4 = 5–6, 6 = >6), most frequently visited health organizations in the past years (1 = provincial hospital or above, 2 = city hospitals, 3 = county hospitals, 4 = township hospitals, 5 = community health service center/village clinic, 6 = self-treatment, 7 = other), family history of epilepsy (1 = yes, 2 = no), being with other chronic disease or nor (1 = yes, 2 = no), annual payment out of insurance for epilepsy (1 = 1999 RMB and below, 2 = 2000–7999 RMB, 3 = 8000 RMB and above), annual personal income (1 ≤ 4999 RMB, 2 = 5000–19,999 RMB, 3 ≥ 20,000 RMB) were entered as independent variables. Step-wise logistic regression was conducted to analyse the risk factors for low HSR using significance levels of 0.05 for entry and 0.10 for removal from the model. Otherwise, the two-tailed significance threshold was set at *p* < 0.05.

## 3. Results

### 3.1. Characteristic of the Study Participants

A total of 420 participants aged 8–86 years (mean = 46.7, SD = 14.32) were enrolled in the study. Males comprised 59.3% of the participants. The majority of participants were farmers (*n* = 339, 80.7%), and had a stable marriage (*n* = 283, 67.4%). The education of the participants was low, 51.7% of them were with an education of primary school and below.

During the past year, more than half (*n* = 240, 57.1%) of the epileptic patients had seizures lower than three times, and 43.1% of the patients preferred to visit county hospitals for health service utilization. Few participants (*n* = 20, 4.8%) had other chronic diseases, and only 5 (1.2%) participants had a family history of epilepsy. A total of 380 (90.7%) participants had an annual payment out of insurance for epilepsy lower than 1999 RMB, and 164 (39.1%) participants had an annual personal income lower than 20,000 RMB (Table 1).

### 3.2. Level of Patient-Perceived HSR of EMP in Rural China 

From epilepsy patients’ responses to eight domains of HSR of EMP, the majority of them had higher evaluation on the performance of dignity (*n* = 383, 91.2%) and confidentiality (*n* = 381, 90.7%). However, they had the lowest evaluation on the performance of social support (*n* = 331, 78.8%) and the choice of providers (*n* = 326, 77.6%) (Table 2).

According to the scores of patients-perceived HSR, the descending order of eight domains of patients-perceived HSR were confidentiality, dignity, choice of providers, autonomy, communication, prompt attention, social support, and basic amenities. Among the eight domains, confidentiality had the highest score as an indicator of HSR (8.93 out of a maximum of 10.0) and was ranked as “Confidentiality of Always and Often” by 90.7% of the respondents, suggesting that the EMP had achieved good results in meeting patients’ confidentiality expectations. However, scores for social support, basic amenities, and prompt attention were lower than other domains, which indicated that these domains should be paid more attention. (Table 3).

Meanwhile, the overall average score of the patients-perceived HSR of EMP was 8.632. This was calculated by the formula according to WHO as follows [8]:

Y = V1 × 0.125 + V2 × 0.125 + V3 × 0.125 +V4 × 0.125 + V5 × 0.200+ V60 × 0.100 + V7 × 0.150 + V8 × 0.050 = 8.74 × 0.125 + 8.64 × 0.125 + 8.93 × 0.125 + 8.61 × 0.125 + 8.53 × 0.200 + 8.41 × 0.100 + 8.38 × 0.150 + 8.66 × 0.050 = 8.632.

### 3.3. Distribution of Patient-Perceived HSR in Rural China

According to the responses from the vulnerable participants (female, elderly, low education, low income, farmer) and the REI (REI = 0.732), the distribution of patients-perceived HSR in the Epilepsy Management Project in rural China was fair. The responses of never being treated unfairly varied from 77.8% to 87.1% (Table 4).

### 3.4. Related Factors of Low Patients-Perceived HSR Scores

Table 5 and Table 6 show the risk factors for low HSR scores. Female, old age, and high education were significantly related to the lower HSR scores of the rural Epilepsy Management Project (Table 5 and Table 6). 

## 4. Discussion

This is the first study to explore the patient-perceived HSR of the Epilepsy Management Project in rural China. This present study revealed the level, distribution, and relevant factors of the HSR of the EMP in rural Hunan province of China as perceived by patients during the period of COVID-19, which has given a clearer picture of the HSR.

This study found that the responsiveness of the EMP in rural Hunan of China achieved a score of 8.632, which indicated a satisfactory level. According to other domestic reports, the responsiveness of the EMP in rural Hunan was higher than those of Shangdong Province (7.13) [30], Shanghai City (8.13) [31], Wuhan City (7.46) [17], Shenzhen City (6.83) [32], Fuzhou City (4.72) [33]. In 2000, China ranked 88th with a responsive score of 7.33 [27], which was lower than that of this study, suggesting that there was an obvious improvement in the health system in China. In terms of responsiveness distribution, the REI of the EMP in rural Hunan was 0.732, which also indicated a relatively balanced distribution of responsiveness. This was consistent with other studies. Yan Y et al. reported that 95.2% of the residents were satisfied with the overall quality of the CHSs in Wuhan City [34].

As is known to all, the COVID-19 brings challenges to many countries, including China. From one previous study of ours [35], the good compliance of epilepsy patients in the Epilepsy Management Project in a rural area of China was reported from 2017 to 2019. Thus, we conducted this study to explore the HSR of the Epilepsy Management Project in Rural China during the period of COVID-19, to find out whether the COVID-19 had an impact on the Epilepsy Management Project in rural China. In a conclusion, owing to the good HSR, this study has given evidence that the Epilepsy Management Project in Rural China is with the ability to serve patients even under crises such as COVID-19, and the Epilepsy Management Project in Rural China should be generalization widely in China.

Among the eight domains of patients-perceived HSR of the EMP in rural Hunan province, confidentiality and dignity were ranked highest and social support and basic amenities lowest. However, the rating of HSR varied in different studies. In Wuhan City of China [17,34] and Israel [36], dignity and confidentiality ranked highest, but the choice and prompt attention ranked lowest. In Anhui Province of China [37], dignity ranked highest and choice lowest. In Shandong Province [30], autonomy and choices ranked lowest, whereas dignity, prompt attention, and basic amenities were lowest in Shenzhen City [32]. In South Africa, the three domains with the lowest ranking were prompt attention, autonomy, and basic amenities [38]. Similarly, in the results of 35 counties reported by the WHO, social support and confidentiality ranked highest and autonomy and basic amenities lowest [28]. In 16 OECD counties, the two highest domains were choice and dignity, and the two lowest domains were prompt attention and communication [39]. For European countries, social support was the best-performing domain of HSR [40]. Among our study population, patients in rural EMP were needed to maintain good relationships for providing free medicine and follow-up regularly, so confidentiality and dignity may be paid obviously attention. On the contrary, social support and basic amenities had fewer supplements due to the insufficient condition in rural areas. Given that the health services needs of our study patients in rural areas were relatively met by the existing medical institutions in their respective prefectures, only rarely was required from other hospitals or medical institutions. Thus, our results revealed that two important determinants of responsiveness, social support, and basic amenities, required improvement in the rural EMP of Hunan Province surveyed.

It is known to all, responsiveness is more sensitive to demographic factors. Recognition of the relations between the characteristics of the population and the preference for healthcare system services will help healthcare officials to determine needs accordingly and to set priorities. However, the factors that affect the responsiveness have been inconclusive so far. Our findings reveal that female, old age, and low education were independent risk factors for low patients-perceived HSR of the EMP during the COVID-19 period in rural Hunan province, which was similar to previous studies. Participants who require high health services have higher expectations of HSR. NIoH et al. found that the growth of people’s expectations and attention to safety, quality, and justice has increased obviously recent years [41], such as the increasing demand for convenience for the elderly to receive medical treatment, higher expectations about the medical environment, prompt attention and so on. A previous study showed that females and the elderly have a great demand for health services, which also affects the overall responsiveness [42]. For example, Ughasoro et al. found that women gave lower scores to the quality of basic amenities [43], which was in accordance with our study.

In agreement with other studies, the level of HSR was varied by educational attainment in the EMP [44,45,46]. Among the participants of our study, many of them were with low education (51.7% of them were with the education of primary school and below). According to the results of our study, the higher education, the more scores of HSR. The differences included eight domains of the HSR in the EMP (Table 5). Chetna et al. reported that the highest education was more likely to report “very good” responsiveness whether public or private health facilities [47,48]. This might be because more educated persons received more information than those who were educated less [49]. As Ergler et al. reported, physicians prefer to provide less medical information for the doubt of a poor person’s ability to understand medical information [46], those less educated may be treated with less understanding. Thus, efforts to reduce socio-economic disparities in health system responsiveness need to take into account the above reasons for these disparities. In addition, at present, according to the Epilepsy Management Project in rural China, only the convulsive epilepsy patients were selected and only the AEDs treatment was provided in the project. Thus, for many other types of epilepsy patients, treatment options besides AEDs and underlying mechanisms of epilepsy seizure could be added to the EMP to make better patient-perceived HSR [47,48,49].

### Strength and Limitation

China has undergone recent reform to improve the basic medical and health services, as well as the equalization of basic public health services. There main strength of our study is that we attempt to explore the HSR and its relevant factors in the Epilepsy Management Project in rural China during the period of COVID-19, so as to provide advice on the improvement of further strategies. However, there remain several limitations. First, it is limited to patients only, other health service users of the EMP were excluded, such as doctors and family members. Second, a limitation of this study is its cross-sectional design, so causation cannot be inferred. Third, there might be a possibility of recall bias and reporting bias, which could not be avoided for the present design. Fourth, the survey was limited to Hunan province, thereby decreasing the external applicability of the conclusions. Fifth, due to the asymmetric information between health providers and users, the evaluation of HSR might be subjected to the irrational expectation of health users [50]. Then, further studies are needed to consummate in the future.

## 5. Conclusions

The HSR of the Epilepsy Management Project in rural China was fairly good. However, measures to improve the patient-perceived HSR are still needed, especially including better service, higher social support, and more comfortable medical environments. Policymakers should consider monitoring and quality assurance reform strategies that focus on the areas of responsiveness to reduce the gap between patients’ expectations and their experiences with health services.

## Figures and Tables

**Table 1:** Characteristic of the study participants.

Variables	*n*	Percentage (%)
Gender		
Male	249	59.3
Female	171	40.7
Age (years)		
≤39 *	127	30.2
40–59	212	50.5
≥60	81	19.3
Marital status **		
Stable	283	67.4
Unstable	137	32.6
Occupation		
Farmer	339	80.7
Employment	38	9.1
Unemployment	37	8.8
Student	6	1.4
Education level		
Below primary school	63	15
Primary school	154	36.7
Middle school	142	33.8
High school and above.	61	14.5
Number of recurrent seizures in the past year		
0	123	29.3
1–2	117	27.9
3–4	67	16
5–6	39	9.3
>6	74	17.6
Most frequently visited health organizations in the past years		
Provincial hospital or above	24	5.7
City hospitals	31	7.4
County hospitals	181	43.1
Township hospitals	97	23.1
Community health service center/village clinic	34	8.1
Self-treatment	29	6.9
Other	24	5.7
Family history of epilepsy		
Yes	5	1.2
No	415	98.8
Being with other chronic disease or nor		
Yes	20	4.8
No	400	95.2
Annual payment out of insurance for epilepsy (RMB)		
≤1999	380	90.7
2000–7999	33	7.9
≥8000	6	1.4
Annual personal income (RMB)		
≤4999	63	15
5000–19,999	101	24.1
≥20,000	255	60.9

*: Five children epilepsy patients included. **: stable marital status was defined as marriage; while unstable marital status included divorced, widowed or widowed, never married, unmarried cohabitation, and so on.

**Table 2:** Patient-perceived responsiveness of epilepsy care in rural China.

Patient-Perceived HSR Domains	Responses (*n*, %)				
	Always	Usually	Sometimes	Never	Always + Usually
Dignity	230 (54.8)	153 (36.4)	36 (8.6)	1 (0.2)	383 (91.2)
Autonomy	197 (46.9)	164 (39.0)	57 (13.6)	2 (0.5)	361 (85.9)
Confidentiality	262 (62.4)	119 (28.3)	36 (8.6)	3 (0.7)	381 (90.7)
Communication	217 (51.7)	139 (33.1)	63 (1.0)	1 (0.2)	356 (84.8)
Prompt attention	201 (47.9)	161 (38.3)	57 (13.6)	1 (0.2)	356 (84.8)
Social support	165 (39.3)	166 (39.5)	89 (21.2)	0	331 (78.8)
Basic amenities	148 (35.2)	206 (49.0)	65 (15.5)	1 (0.2)	354 (84.2)
Choice of providers	163 (38.8)	163 (38.8)	86 (20.5)	8 (1.9)	326 (77.6)

**Table 3:** Scores of level of patients-perceived HSR.

Domains	Weight *	$\bar{x}\;\pm \;\mathrm{SD}$	Ranking
Dignity	0.125	8.74 ± 1.281	2
Autonomy	0.125	8.64 ± 1.479	4
Confidentiality	0.125	8.93 ± 1.298	1
Communication	0.125	8.61 ± 1.278	5
Prompt attention	0.200	8.53 ± 1.367	6
Social support	0.100	8.41 ± 1.320	7
Basic amenities	0.150	8.38 ± 1.405	8
Choice of providers	0.050	8.66 ± 1.356	3
Total score ^#^		8.632 ± 1.351	

* the weight was from KIS; ^#^ the score was calculated by the formula from KIS.

**Table 4:** Distribution of patient-perceived HSR among disadvantaged groups.

Populations	Responses of Unfair (*n*,%)				
	Always	Often	Sometimes	Never	Total
Female	0	6 (3.5)	16 (9.4)	149 (87.1)	171 (100.0%)
Elderly (≥60 years)	3 (3.7)	4 (4.9)	11 (13.6)	63 (77.8)	81 (100.0%)
Low education (primary school and below)	3 (1.4)	7(3.2)	21 (9.7)	186(85.7)	217 (100.0%)
Low income (≤4999 RMB)	2 (3.2)	5 (7.9)	7 (11.1)	49 (77.8)	63 (100.0%)
Farmer	5 (1.5)	13 (3.8)	53 (15.6)	268 (79.0)	339 (100.0%)

**Table 5:** Comparisons of patients-perceived HSR scores among different demographic characteristics.

Variables	Dignity	Autonomy	Confidentiality	Communication	Prompt Attention	Social Support	Basic Amenities	Choice of Providers
Gender	Z = 7.024 *	Z = 11.935 **	Z = 4.021 *	Z = 4.124 *	Z = 4.771 *	Z = 3.271	Z = 6.315 *	Z = 7.137 *
Age	Z = 8.665 **	Z = 8.575 **	Z = 10.194 **	Z = 9.775 **	Z = 9.745 **	Z = 9.233 **	Z = 9.771 **	Z = 10.225 **
Marital status	Z = 0.173	Z = 0.012	Z = 4.419 *	Z = 0.960	Z = 4.130 *	Z = 2.031	Z = 0.224	Z = 0.919
Occupation	Z = 4.002 *	Z = 1.339	Z = 2.955	Z = 0.002	Z = 0.680	Z = 0.345	Z = 0.682	Z = 4.534 *
Education	Z = 19.448 **	Z = 17.466 **	Z = 10.448 *	Z = 18.401 **	Z = 16.735 **	Z = 14.517 *	Z = 16.038 *	Z = 20.370 **
Number of recurrent seizures	r = −0.037	r = −0.10 *	r = 0.035	r = 0.062	r = 0.004	r = 0.092	r = −0.074	0.099
Number of hospital visited	r = −0.020	r = −0.079 **	r = −0.016	r = −0.053	r = −0.074	r = −0.074 *	r = −0.042	−0.025
Family history	Z = 0.037	Z = 0.003	Z = 2.194	Z = 5.002 *	Z = 5.343 *	Z = 5.078 *	Z = 6.180 *	Z = 0.497
Other chronic disease	Z = 2.352	Z = 3.451	Z = 4.331	Z = 3.159	Z = 5.721 *	Z = 2.076	Z = 5.472 *	Z = 1.675
Annual payment out of insurance for epilepsy	r = −0.144 *	r = −0.082 *	r = −0.219	r = 0.023 *	r = 0.048	r = 0.075 *	r = 0.011 *	r = 0.076 *
Annual personal income	r = −0.161 *	r = −0.131 *	r = −0.050 *	r = −0.090	r = −0.054 *	r = −0.007	r = −0.069 *	r = −0.081

* *p* < 0.05; ** *p* < 0.01.

**Table 6:** Results of binary logistic regression analysis of risk factors for low HSR.

Variables	β Coefficient	Walds Test	*p*	OR	95% CI
Gender					
Male				1.00	
Female	−0.50	5.80	0.016	0.61	(0.40, 0.91)
Age (years)					
≤39				1.00	
40–59	−0.49	5.21	0.023	0.62	(0.41, 0.93)
≥60	−0.50	11.31	0.001	0.61	(0.45, 0.81)
Education					
High school and above				1.00	
Middle school	−0.49	5.98	0.014	0.61	(0.41, 0.91)
Primary school and below	−0.68	11.33	0.001	0.50	(0.34, 0.75)

## Data Availability

The data presented in this study are available on request from the corresponding author.
